# Loss of miR-200c: A Marker of Aggressiveness and Chemoresistance in Female Reproductive Cancers

**DOI:** 10.1155/2010/821717

**Published:** 2009-12-15

**Authors:** Dawn R. Cochrane, Erin N. Howe, Nicole S. Spoelstra, Jennifer K. Richer

**Affiliations:** Department of Pathology, Denver School of Medicine, University of Colorado, Aurora CO, 80045, USA

## Abstract

We focus on unique roles of miR-200c in breast, ovarian, and endometrial cancers. Members of the miR-200 family target ZEB1, a transcription factor which represses E-cadherin and other genes involved in polarity. We demonstrate that the double negative feedback loop between miR-200c and ZEB1 is functional in some, but not all cell lines. Restoration of miR-200c to aggressive cancer cells causes a decrease in migration and invasion. These effects are independent of E-cadherin status. Additionally, we observe that restoration of miR-200c to ovarian cancer cells causes a decrease in adhesion to laminin. We have previously reported that reintroduction of miR-200c to aggressive cells that lack miR-200c expression restores sensitivity to paclitaxel. We now prove that this ability is a result of direct targeting of class III beta-tubulin (TUBB3). Introduction of a TUBB3 expression construct lacking the miR-200c target site into cells transfected with miR-200c mimic results in no change in sensitivity to paclitaxel. Lastly, we observe a decrease in proliferation in cells transfected with miR-200c mimic, and cells where ZEB1 is knocked down stably, demonstrating that the ability of miR-200c to enhance sensitivity to paclitaxel is not due to an increased proliferation rate.

## 1. Introduction

Specific miRNAs have been found to be expressed in cell type-specific manner, at specific developmental stages, and in disease states including cancer [1, 2]. During the initiation and progression of cancer, miRNAs have been observed to act as oncogenes or tumor suppressors [3, 4]. While some miRNAs are overexpressed in cancers, the majority appear to be lost and often localize to fragile sites [5]. Differences in the miRNA expression profiles of normal compared to cancerous tissue of the endometrium, breast and ovary have been documented [6–11]. MiRNAs can affect the expression of a large number of proteins, including those involved in pathways relevant to cancer, such as apoptosis, migration and metastatis. Thus, miRNAs hold promise as biomarkers for several types of cancer [12, 13].

Epithelial to mesenchymal transition (EMT) is a normal process that occurs during development in which individual cells or groups of cells become motile. The same process is thought to be used by cancer cells during tumor progression to enable them to become more motile and thus more metastatic [14]. EMT involves reprogramming of the cells by transcription factors such as ZEB1, SIP1 (ZEB2), Twist, Snail, and Slug [15]. A hallmark of EMT is loss of E-cadherin expression, loss of polarity, acquisition of mesenchymal markers, and increased motility [16, 17]. Both ZEB1 and the closely related ZEB2 bind E-box like sequences in the E-cadherin promoter, recruit the corepressor CtBP and thereby repress E-cadherin [18]. ZEB1 also represses additional genes involved in polarity [16]. ZEB1 expression is confined to cells of mesenchymal origin, while normal epithelial cells and low grade carcinomas do not express ZEB1. However, we and others have shown that in high grade, aggressive carcinomas that have undergone EMT, ZEB1 can be expressed, leading to loss of E-cadherin [19–22].

Several miRNAs have been implicated in the process of EMT, among them are the members miR-200 family [23–25]. This family contains five members (miR-200a, -200b, -200c, -141 and, -429) which are highly homologous. Originally, miR-200c was reported to directly bind ZEB1 and cause degradation of the mRNA, resulting in an upregulation of E-cadherin [26]. Subsequently, other reports have shown that all members of the miR-200 family, since they share a high degree of homology especially in their seed sequence, are capable of repressing both ZEB1 and ZEB2 [27–29]. We have demonstrated that miR-200c represses not only ZEB1/2, but a program of transcripts normally expressed only in cells of mesenchymal origin [30]. Since members of the miR-200 family are responsible for repressing ZEB1 and ZEB2 as well as other mesenchymal genes, these miRNAs are considered “guardians of the epithelial phenotype.” MiR-200 family members are therefore thought to be expressed in an epithelial cell-specific manner in normal tissues. Recently, the ability of ZEB1 to transcriptionally repress expression of miR-200 family members has been documented [31, 32]. This double negative feedback loop between miR-200 family members and ZEB1 allows for plasticity between the epithelial and mesenchymal states [33].

In this paper, we focus on the role of miR-200c in breast, ovarian, and endometrial cancers. The mutual repression between ZEB1 and miR-200c is functional in some, but not all cells that we have tested. Increasing miR-200c levels causes a decrease in adhesion to laminin. We demonstrate that the decrease in migration and invasion observed when miR-200c is reintroduced to cancer cells that lack it is independent of restoration of E-cadherin. Lastly, we have previously demonstrated that class III beta tubulin (TUBB3) is directly controlled by miR-200c. Expression of TUBB3 is known to be a common mechanism of resistance to microtubule-targeting agents in many types of cancer. Here, we present conclusive data that repression of TUBB3 is the mechanism whereby miR-200c restores sensitivity to paclitaxel. Taken together, these data demonstrate that loss of miR-200c is a marker for chemoresistance and aggressiveness in breast, ovarian, and endometrial cancers.

## 2. Materials and Methods

### 2.1. Cell Culture

Hec50 cells, representing the more aggressive Type II endometrial cancers [19], were cultured in DMEM with 10% FBS, and 2 mM L-glutamine. MDA-MB-231 are a triple negative breast cancer cell line and were grown in media containing 5% FBS, HEPES, nonessential amino acids, L-glutamine, penicillin, streptomycin, and insulin. Hey cells were grown in RPMI with 5% FBS. All cells were grown in a 37°C incubator with 5% CO_2_. The identity of all the cell lines was confirmed by DNA profiling using the Identifiler Kit (Applied Biosystems).

### 2.2. Transfections

Lipofectamine 2000 (Invitrogen) was combined with pre-200c (miRNA mimic) or scrambled negative control (Ambion) at a concentration of 60 nM and incubated in serum free RPMI for 20 minutes prior to addition to Hey cells. Cells were incubated at 37°C for 4 hours before replacement of FBS to 10%. Protein and RNA were harvested 48 hours posttransfection.

TUBB3 (from Fernando Cabral, University of Texas - Houston Medical School) was cloned into pCI-neo. Transient transfection of 3.3 *μ*g of TUBB3 plasmid or empty vector (pCI-neo, Promega) per well in a 6-well plate was performed using lipofectamine 2000.

### 2.3. Generation of Stable Cell Lines

Transduction of cells was performed using SMARTvectorTM shRNA Lentivral Particles (Thermo Scientific Dharmacon). Each cell line was transduced with 3 separate lentiviral constructs targeting ZEB1 as well as two controls: SMARTvector Empty Vector particles and SMARTvector Firefly Luciferase Control particles. The former is a negative control and does not correlate with gene silencing and the latter is a positive control targeting firefly luciferase plasmids PGL2 and PGL3. All vectors are packaged and contain a TurboGFP and an SCMV promoter, as well as a puromycin-resistance selectable marker.

MDA231 and Hec50 cells were plated at 3000 cells/well and 1500 cells/well, respectively, in triplicate using 96 well plates. The following day, media was replaced with 80 *μ*l of fresh media containing 10 *μ*g/mL polybrene (Sigma). The amount of viral particles/well was determined using the following calculation: (MOI × CN)/VT, where MOI (multiplicity of infection) = 10 TU/cell, CN = number of cells/well, and VT = stock viral titer of 10^4^ TU/*μ*L. Viral particles were added in a total volume of 20 *μ*L to each well. The following day, transduction media was removed and wells were rinsed with PBS and replaced with regular media. Once confluent, cells were trypsinized and replated in 48 well plates. At this point, antibiotic selection was initiated and cells were ultimately expanded and maintained using 1 *μ*g/mL of puromycin (Sigma).

### 2.4. Immunoblotting

Whole cell protein extracts were denatured and 50 *μ*g separated on 8% SDS PAGE gels and transferred to PVDF membranes. The membranes were blocked in 5% milk in TBS-T, and then probed overnight at 4°C. Primary antibodies were diluted in 5% milk in TBS-T. The primary antibodies used were ZEB1 (rabbit polyclonal from Dr. Doug Darling, University of Kentucky, 1 : 1500 dilution), E-cadherin (clone NCH-38 from DAKO, 1 *μ*g/mL), TUBB3 (rabbit polyclonal PRB-435P from Covance, 1 : 5000 dilution), and *α*-tubulin (clone B-5-1-2 from Sigma, 1 : 20000 dilution). After incubation with appropriate HRP-conjugated secondary antibody, bands were detected using Western Lightning Chemiluminescence Reagent Plus (Perkin Elmer).

### 2.5. Real Time RT-PCR

RNA was harvested from cells using Trizol (Invitrogen) as per the manufacturer's instructions. Prior to generating cDNA, mRNA was treated with DNase1 (Invitrogen) for 15 minutes at room temperature. RNA was reverse transcribed into cDNA in a reaction containing reaction buffer, dNTPs, RNAse inhibitor (Applied Biosystems), random hexamers, and 200 U of MuLV-RT (Applied Biosystems). The reaction proceeded at 22°C for 10 minutes, then at 37°C for one hour. For normalization, real time RT-PCR was performed on the cDNA using eukaryotic 18S rRNA endogenous control primers and FAM-MGB probe (Applied Biosystems). TaqMan MicroRNA Reverse Transcription kit was used to generate cDNA for real time RT-PCR reaction in conjunction with a miR-200c specific primer and probe (ABI, assay ID 002300). The reverse transcription primer for miR-200c is a hairpin primer which is specific for the mature miRNA and will not bind to the precursor molecules. Reported values are the means and standard errors of 3 biological replicates.

The relative mRNA or miRNA levels were calculated using the comparative Ct method (ΔΔCt). Briefly, the cycle threshold (Ct) values for the rRNA were subtracted from Ct values of the target gene to achieve the ΔCt value. The 2^−ΔCt^ was calculated for each sample and then each of the values was then divided by a control sample to achieve the relative miRNA levels (ΔΔCt).

### 2.6. Migration and Invasion Assays

The assays were performed on MDA-MB-231 stable empty vector or shZEB1 #2, or Hey cells transiently transfected with the miR-200c mimic for 48 hours. Cells were serum starved for 12 hours prior to performing the assay. BD BioCoat Control Insert Chambers 24-well plate with 8 micron pore size and BD BioCoat Matrigel Invasion Chambers were used for migration and invasion assays, respectively. After starvation, cells were removed from the plate and 50000 Hey cells or 250000 MDA-MB-231 cells were plated in 0.5 mL media with 0.5% FBS in the upper chamber. In the lower chamber 0.8 mL of 50% conditioned media plus 50% complete media containing an additional 10% FBS was used as an attractant. Hey cells were incubated for 24 hours and MDA-MB-231 cells for 48 hours at 37°C. Migrating or invading cells on the lower surface of the membranes were stained with Diff-Quik stain (Fisher) and counted manually using ImagePro Plus software (Mediacybernetics Inc.).

### 2.7. Adhesion Assays

Adhesion assays were performed using InnoCyte ECM Cell Adhesion Assays (Calbiochem) for Collagen IV, Fibronectin, Basement Membrane Complex and Laminin. To each well 50000 cells were added and the plates were incubated at 37°C for 1.5 hour. The wells were gently washed with PBS before adding the Calcein-AM solution. The plates were incubated for 1 hour at 37°C, and fluorescence was read with an excitation wavelength of 485 nm and an emission wavelength of 528 nm. The relative fluorescent units were plotted, and the error bars represent standard error of the mean over four replicates.

### 2.8. Clonogenic Assay

Hey cells were plated into 6-well plates at a density of 2000 cells per well. Twenty-four hours after plating, the cells were treated with 0, 1, 2, 3, 4, or 5 nM paclitaxel (Sigma) in triplicate. The cells were incubated at 37°C for 8 days before fixing and staining with crystal violet. Photos were taken of the plates and the images analyzed using ImageJ software (NIH). The average number of colonies and the average total area was plotted, with error bars representing the standard error of the mean over the three replicates.

### 2.9. Cell Death ELISAs

Hec50 cells were transfected with the miR-200c mimic as described previously for the Hey cells. Twenty-four hours after transfection, cells were treated with 0, 15, 20 or 25 nM paclitaxel (Sigma). Twenty-four hours after treatment, the Cell Death ELISA (Roche) which recognizes mono- and oligonucleosomes in the cytoplasm of dying cells was performed as per manufacturer's instructions.

### 2.10. Proliferation Assays

Cells were plated into 6-well plates (4000 Hey cells/well and 6000 MDA-MB-231 or Hec50 cells/well). At time points indicated, cells were trypsinized and counted using the Vi-cell Cell Viability Counter (Beckman Coulter).

## 3. Results

### 3.1. Reciprocal Repression of miR-200c and ZEB1 Occurs in Some but Not All Cell Types

**Figure 1:**
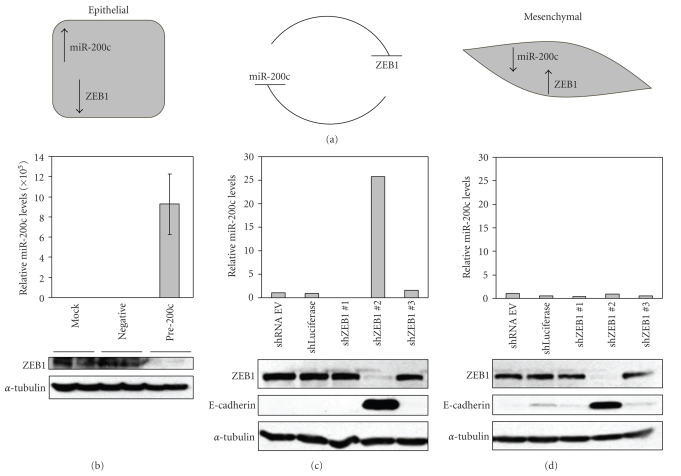
Reciprocal repression between ZEB1 and miR-200c occurs in some, but not all cell types. (a) Schematic of the mutual repression of ZEB1 and miR-200c. (b) Western blotting for ZEB1 and *α*-tubulin loading control, and real time RT-PCR for miR-200c in Hey ovarian cells transiently transfected with a miR-200c mimic. Real time RT-PCR for miR-200c and western blotting for ZEB1, E-cadherin, and *α*-tubulin in MDA-MB-231 breast cancer cells (c), and Hec50 endometrial cancer cells (d) stably transfected with shRNA lentiviral vector targeting ZEB1 (shZEB), luciferase (shLuciferase), or the empty vector (shRNA EV).

**Figure 2:**
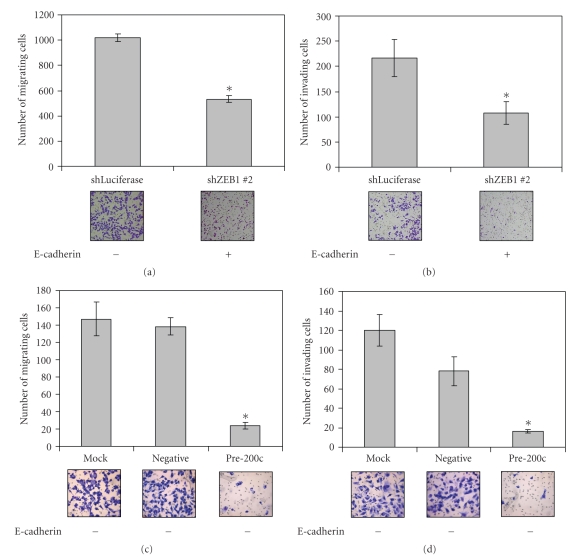
Increased miR-200c decreases migration and invasion, not necessarily dependent on restoration of E-cadherin. Migration (a) and invasion (b) assays for MDA-MB-231 cells stably expressing an shRNA targeting ZEB1 with representative images below. Migration (c) and invasion (d) assays in Hey cells transiently transfected with a miR-200c mimic. Asterisks indicate a statistically significant difference (*P* < .05, Student's *t*-test) versus negative controls.

Just as members of the miR-200 family can repress ZEB1 by degradation of its transcript, ZEB1 can repress expression of the miR-200 family members by binding to E-boxes within their promoter regions [31, 32]; see Figure 1(a). Directly increasing miR-200c levels in Hey cells (aggressive serous ovarian cell line) by transfection of a miR-200c mimic (pre-200c) results in repression of ZEB1 expression (Figure 1(b)). Although ZEB1 is a repressor of E-cadherin, we did not observe E-cadherin expression induced by the repression of ZEB1 in these cells (data not shown). However there are several other mechanisms through which E-cadherin can be lost including methylation of the promoter [34, 35] and chromosomal deletion [36, 37]. In contrast, we have previously shown that transient transfection of the miR-200c mimic into MDA-MB-231 (an aggressive triple negative breast cancer cell line) and Hec50 (an aggressive Type 1 endometrial cancer cell line) causes a marked repression of ZEB1 and a restoration of E-cadherin expression [30]. Presently, we stably transduced lentiviral shRNAs targeting ZEB1 into these two cell lines (Hec50 and MDA-MB-231). While two of the shRNAs did not decrease ZEB1 protein, shRNA #2 caused an almost complete repression of ZEB1 expression resulting in re-expression of E-cadherin in both cell lines (Figures 2(c) and 2(d)). Intriguingly, while knock down of ZEB1 in MDA-MB-231 cells causes the expected increase in miR-200c levels (indicative of the reciprocal regulation), no such increase is observed in Hec50 cells. This suggests that while reciprocal repression of miR-200c and ZEB1 occurs in some cell lines, it does not occur in all.

### 3.2. Restoration of miR-200c to Aggressive Cancer Cells Results in Decreased Migration, Invasion, and Cell Adhesion

It has been previously shown that the miR-200 family members cause a decrease in cell migration and invasion [27, 29, 30]. We observe a decrease in migration and invasion in the MDA-MB-231 cells in which ZEB1 has been knocked down, resulting in an increase in miR-200c levels. In the MDA-MB-231 cells there is 52% decrease in migration and a 50% decrease in invasion in the shZEB1 #2 containing cells in which ZEB1 is completely knocked down versus luciferase control (Figures 2(a) and 2(b)). We show here that the same holds true in the aggressive ovarian cancer Hey cell line. This cell line is highly migratory and invasive, and reintroduction of miR-200c to these cells results in an 83% decrease in migration and a 79–86% decrease in invasion compared to negative controls (Figures 2(c) and 2(d)). However, it is interesting to note that the effect on migration and invasion caused by miR-200c is independent of the E-cadherin status of the cells, since unlike the MDA-MB-231 cells, Hey cells do not regain E-cadherin expression in response to decreased ZEB1 levels.

**Figure 3:**
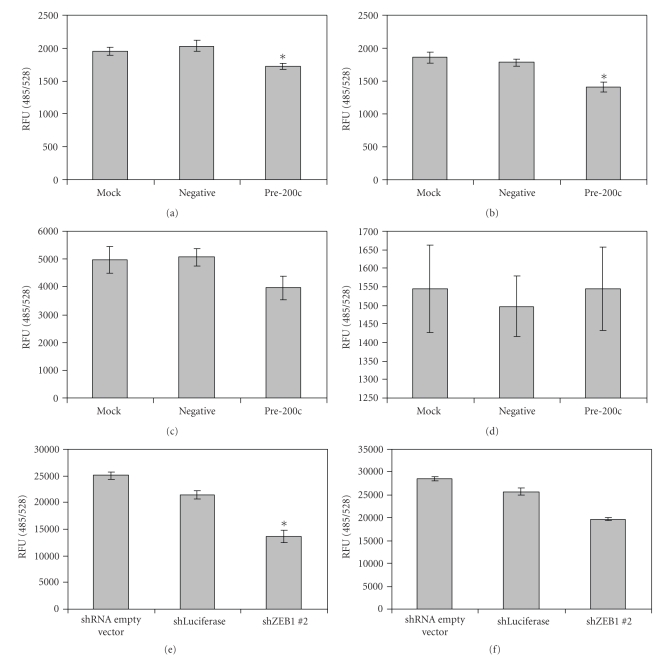
Increased miR-200c levels decrease adhesion to substrates. Fluorescent adhesion assays of Hey cells to (a) basement membrane complex, (b) laminin, (c) collagen type IV, and (d) fibronectin. Adhesion of MDA-MB-231 cells to (e) basement membrane complex and (f) laminin. Asterisks indicate a statistically significant difference (*P * < .05, Student's *t*-test) versus the negative controls.

While E-cadherin protein affects epithelial cell-cell contact, we also wished to determine if miR-200c affects adhesion to substrates as measured by fluorescent adhesion assays. Hey cells transiently transfected with the miR-200c mimic showed a small but statistically significant decrease in adhesion to basement membrane complex (BMC) and laminin (Figures 3(a) and 3(b)). There is also a trend towards decreased adhesion to collagen IV (Figure 3(c)); however, this did not reach statistical significance. No difference in adhesion to fibronectin was observed (Figure 3(d)). Since there was an affect on adhesion to BMC and laminin in ovarian cancer cells with high miR-200c levels, we performed the adhesion assay with the Hec50 and MDA-MB-231 cells in which ZEB1 had been stably knocked down. We again see a decrease in adhesion to BMC and laminin in the MDA-MB-231 cells; however, only the decrease in BMC binding is statistically significant (Figures 3(e) and 3(f)). In contrast to the Hey and MDA-MB-231 cells, there was no decrease in adhesion to either substrate in the Hec50 cells in which ZEB1 is knocked down (data not shown), but there is not a concomitant increase in miR-200c, as shown in Figure 1(d). This result suggests that the effects on adhesion may be mediated through miR-200c.

### 3.3. Increased Chemosensitivity to Paclitaxel with miR-200c Expression

**Figure 4:**
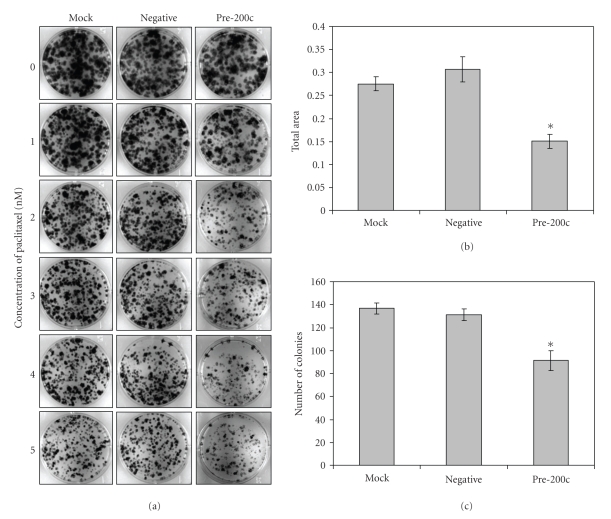
Chemosensitivity to paclitaxel induced by miR-200c expression. (a) Clonogenic assay in Hey cells either mock transfected, transiently transfected with a negative control, or miR-200c mimic and treated with 0–5 nM paclitaxel. The total area (b) and number of colonies (c) at 5 nM paclitaxel are quantified. Asterisks indicate statistically significant difference (*P * < .05, Student's *t*-test) versus negative controls.

We have previously demonstrated that miR-200c expression causes increased chemosensitivity to microtubule targeting agents such as paclitaxel. While the ELISA cell death assay that we have used previously to demonstrate this property of miR-200c is a short-term assay, we confirm here, in a relatively long-term clonogenic assay, that there is increased sensitivity of Hey cells to paclitaxel when transfected with pre-200c (Figure 4(a)). We observe a 49–55% decrease in total area and a 67–70% decrease in the number of colonies in the pre-200c treated cells versus the negative control with 5 nM paclitaxel treatment (Figures 4(b) and 4(c)). As the assay is conducted over a relatively long period of time, the maximum dose of paclitaxel used is relatively small compared to what is used in the assays that look at acute toxicity (i.e., 24 hours). At doses of paclitaxel of 10 nM and higher, no colonies are formed in the assay (data not shown).

**Figure 5:**
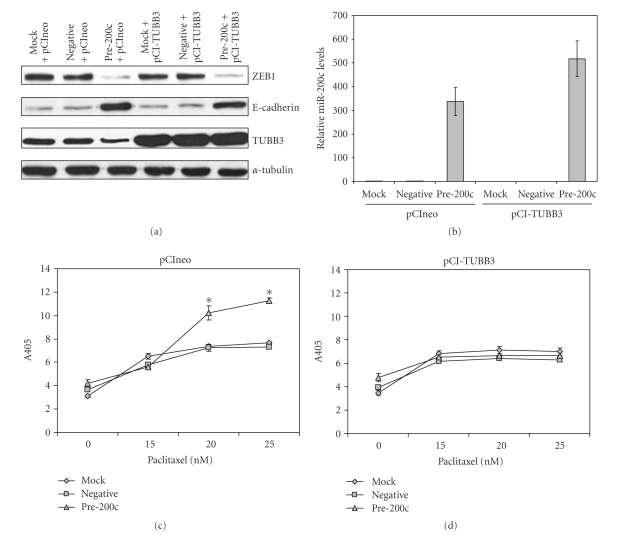
Restoration of TUBB3 reverses miR-200c-mediated enhanced chemosensitivity to paclitaxel. (a) Western blots for ZEB1, E-cadherin, TUBB3, and *α*-tubulin in Hec50 cells transiently transfected with a miR-200c mimic or negative controls and an expression vector for TUBB3 or empty vector. (b) Real time RT-PCR for miR-200c. Cell death ELISA for cells transfected with a miR-200c mimic and an empty vector (c) or TUBB3 expression vector (d) treated with various concentrations of paclitaxel. Asterisks indicate statistically significant difference (*P * < .05, Student's *t*-test) versus negative controls.

We have previously implicated the ability of miR-200c to directly target *TUBB3 * (class III beta tubulin) as being the mechanism responsible for the increased chemosensitivity to microtubule targeting agents. TUBB3 is normally only expressed in neuronal cells; however aberrant expression of TUBB3 in several different types of cancers has been shown to cause resistance to paclitaxel [38–43]. We demonstrated that miR-200c directly targets *TUBB3 * for degradation. To definitively test whether TUBB3 is responsible for the miR-200c-mediated increase in chemosensitivity to paclitaxel, we transfected cells with a TUBB3 construct lacking its 3’ UTR (containing the miR-200c binding site) which is therefore not able to be targeted by miR-200c. Transfection of this exogenous TUBB3 construct does not affect the transfection of the miR-200c mimic, nor its ability to downregulate ZEB1 and upregulate E-cadherin (Figures 5(a) and 5(b)) in Hec50 cells. When the Hec50 cells are transfected with an empty vector (no exogenous TUBB3) in addition to miR-200c mimic, there is a statistically significant increase in sensitivity to paclitaxel as measured in a cell death ELISA; see Figure 5(c). However, when the cells are transfected with the TUBB3 expression vector lacking its 3’ UTR, the enhanced sensitivity to paclitaxel is lost; see Figure 5(d). Therefore, expression of exogenous TUBB3 lacking the miR-200c target site reverses the chemosensitivity to paclitaxel caused by increased miR-200c expression.

**Figure 6:**
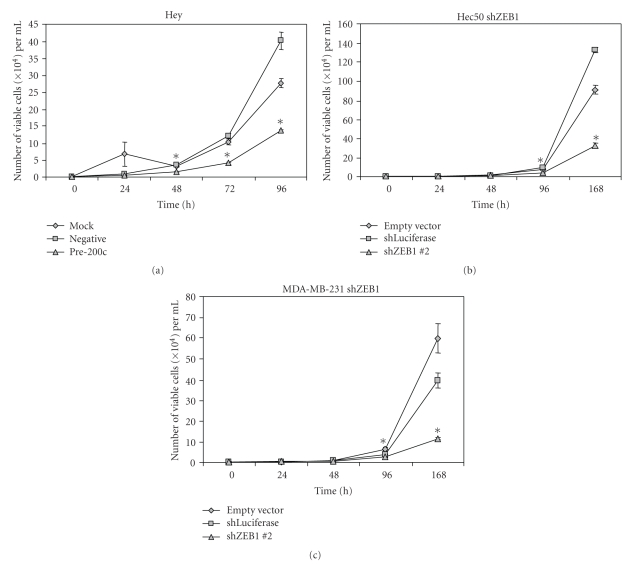
Proliferation assay in Hey cells transiently transfected with a miR-200c mimic (a), or Hec50 cells (b), and MDA-MB-231 cells (c) stably expressing an shRNA against ZEB1. Asterisks indicate statistically significant difference (*P * < .05, Student's *t*-test) versus negative controls.

It can be argued that cells with increased proliferation would be more sensitive to microtubule poisons and that could be an alternative explanation for the observed chemosensitivity upon restoration of miR-200c. We therefore performed proliferation assays in the three cell types and found decreased proliferation in all three (Figures 6(a), 6(b), and 6(c)). Since the decrease in proliferation is observed in all three cell types, including the Hec50s where there was no increase in miR-200c levels, it is likely that the effects on proliferation occur via ZEB1 and not by miR-200c. The fact that the increase in chemosensitivity is found in cells that are proliferating more slowly than the negative controls demonstrates that increased proliferation is not the mechanism behind the increase in chemosensitivity.

## 4. Discussion

In this paper we build on our previous work to further characterize the role that loss of miR-200c plays in generating an aggressive cancer phenotype. We focus on ovarian, endometrial, and breast cancer. The miR-200 family is crucial for the maintenance of the epithelial phenotype. ZEB1 is normally only expressed in cells of mesenchymal origin; however, its aberrant expression is observed in cancers that have undergone EMT. ZEB1 (and the closely related ZEB2) transcripts are targeted by miR-200c and the other miR-200 family members. Interaction of any of the miR-200 family members with the ZEB transcripts results in degradation and inhibition of translation. Therefore the maintenance of miR-200c expression in normal epithelial cells serves to prevent ZEB1 and ZEB2 from being expressed. Since both ZEB1 and ZEB2 repress genes involved in polarity, repression of these proteins serves to maintain polarity, an important epithelial cell characteristic. We have recently shown that in addition to repressing ZEB1 and 2, miR-200c represses a program of transcripts normally only expressed in cells of mesenchymal and neuronal origin, such as fibronectin (FN1), neurotrophic tyrosine kinase (NTRK2), quaking 1 (QKI), and TUBB3 [30]. Thus, miR-200c maintains epithelial cell characteristics not only by maintaining polarity via repression of ZEB1 and ZEB2, but also by repressing additional non-epithelial genes. 

It has been recently demonstrated that miR-200c and ZEB1 regulate each other in a double-negative feedback loop [31, 32]. The miR-200 family of miRNAs is expressed in two clusters, one on choromosome 1p36.33 and the other on chromosome 12p12.31. E-boxes are located in the promoter region of each of these clusters. ZEB1 can bind these E-boxes and directly repress all miR-200 family members [31]. Therefore, in cells that have undergone EMT, ZEB1 and 2 not only serve to repress genes involved in polarity, but also repress the miR-200 family and thereby release the repression of many genes characteristic of the mesenchymal phenotype. Central to the double feedback loop between miR-200c and ZEB1 is TGF-*β*. During TGF-*β *-induced EMT, there is an increase in Ets1 which binds to and activates the promoter of ZEB1 [44]. Therefore, in a tumor microenvironment, increased TGF-*β* levels are thought to result in an increase of ZEB1 transcription to a point where it can overcome the repression caused by miR-200c. As ZEB1 protein begins to be made, it can then repress the miR-200 family members, resulting in progression through EMT [32].

We have previously shown that restoration of miR-200c in Hec50 endometrial cells and MDA-MB-231 breast cancer cells causes repression of ZEB1 and re-expression of E-cadherin protein. Here we show that transfection of miR-200c mimic into Hey cells, an aggressive serous ovarian cell line, also causes a dramatic repression of ZEB1; however no expression of E-cadherin was observed. To test whether the double-negative feedback loop is intact in the Hec50 and MDA-MB-231 cells, these cells were infected with lentivirus expressing an shRNA against ZEB1. In both cell lines, efficient knock down of ZEB1 was achieved, as was re-expression of E-cadherin. In MDA-MB-231 cells, ZEB1 knock down resulted in an increase in miR-200c levels, as would be expected from the negative feedback loop. However, this was not the case in Hec50 cells, where there was no increase in miR-200c. Whether the break in the negative feedback loop is an anomaly of this particular cell line remains to be tested. The mechanism behind the phenomenon is also unknown; however, it does offer an opportunity to dissect the contribution of ZEB1 versus that of miR-200c to the phenotype of the cells. For example, significantly decreased proliferation was observed in the Hey cells transiently transfected with the miR-200c mimic as well as in the MDA-MB-231 and Hec50 cells that have ZEB1 stably knocked down, although miR-200c levels did not rise in the Hec50s. Therefore it is likely that the decrease in proliferation is due to the lack of ZEB1, not an increase in miR-200c. Conversely, the decrease in adhesion to the basement membrane complex and laminin was only observed in the Hey and MDA-MB-231 cells, and not the Hec50s, suggesting that this phenotype is a function of miR-200c expression rather than ZEB1.

We and others have previously shown that restoration of miR-200c to cancer cells that do not express it causes a decrease in invasion and migration [27, 30]. Here we show that knock down of ZEB1 in MDA-MB-231 cells, which causes an increase in miR-200c, negatively affects migration and invasion. Furthermore, we show that restoration of miR-200c in Hey ovarian cancer cells results in a dramatic decrease in migration and invasion even though E-cadherin is not restored in these cells, despite complete repression of ZEB1. Loss of E-cadherin expression can result from mechanisms other than ZEB1 transcription repression, including chromosomal deletion and promoter hypermethylation [34–37]. Possibilities for its continued absence in these cells include promoter methylation such that even when repression by ZEB1 is relieved, E-cadherin will not be expressed, or perhaps levels of another transcriptional repressor such as Snail or Twist remain high and repress E-cadherin. Regardless of the mechanism, the effects of miR-200c on invasion and migration appear to be independent of E-cadherin status. While E-cadherin is involved in epithelial cell-cell adhesion and its expression has been shown to negatively affect migration and invasion [45, 46], increased miR-200c is able to decrease migration and invasion on its own. We have previously observed that restoration of miR-200c affects genes involved in cell motility and invasion such as ARHGDIB, NTRK2, EPHB1, and FN1 [30].

We demonstrate that miR-200c causes a decrease in adhesion to basement membrane complex, laminin, and perhaps collagen type IV. This observation is particularly relevant to ovarian cancer because the cancerous cells adhere to sites within the peritoneal cavity. During the progression of cancer there is switching of the expression patterns of the cell surface adhesion molecules, such as the cadherins and integrins [47–49]. Although the change in the number of adherent cells appears modest, this might play a significant role in developing a potential treatment for ovarian cancer. The ability of ovarian cancer cells to spread and adhere to the peritoneal cavity is one of the major phenotypes of this disease. A small change in ability of the cells to adhere might reflect a great decrease in the tumor burden and/or increase the ability to debulk the tumor. These results are independent of E-cadherin expression since the decrease in adhesion is observed in both the MDA-MB-231 cells (where E-cadherin expression is regained with increased miR-200c) and in Hey cells, where it is not. 

Clonogenic assays reveal that there is an increase in chemosensitivity to paclitaxel with increased miR-200c levels. Indeed, acquired resistance to paclitaxel in ovarian cancer cells has been shown to be associated with EMT, resulting in an aggressive phenotype [50]. Clinically, aberrant expression of TUBB3 (not normally expressed in epithelial cells) has been found to be associated with resistance to taxanes [38–41]. We have previously shown that TUBB3 is a direct target of miR-200c and suggested that its repression by miR-200c is the mechanism behind the ability of miR-200c to increase chemosensitivity to microtubule targeting agents [30]. Here, we perform the definitive experiment to prove that miR-200c-mediated TUBB3 downregulation is indeed the cause of the enhanced chemosensitivity. We utilized exogenous TUBB3 lacking its 3’UTR such that it cannot be targeted by miR-200c and show that resistance to paclitaxel is maintained even in the presence of miR-200c. In contrast, endogenous TUBB3 is reduced when miR-200c is added, resulting in enhanced chemosensitivity to paclitaxel.

Microtubule targeting agents such as paclitaxel work more efficiently in cells that are rapidly dividing. Consequently, it could be argued that the increase in chemosensitivity caused by miR-200c is due to increased proliferation. However, we show that increase of miR-200c or direct knockdown of ZEB1 results in decreased proliferation in three different types of cancer cells. It is therefore the downregulation of TUBB3, not an increase in proliferation that is responsible for the enhanced chemosensitivity to taxanes observed with restoration of miR-200c to resistant cancer cells.

## 5. Conclusions

MiR-200c expression serves to maintain the epithelial phenotype in well-differentiated, low-grade, breast, ovarian, and endometrial cancer cells. This phenotype includes decreased adhesion to laminin and decreased migration and invasion. Furthermore we find that not all of miR-200c's actions can be attributed to the restoration of E-cadherin via targeting of ZEB1. We further prove that miR-200c-mediated repression of TUBB3 is the cause of enhanced chemosensitivity to microtubule targeting agents. Lastly we demonstrate that not all cells exhibit the double negative feedback loop between miR-200c and ZEB1 and that this can be exploited to identify the distinct roles of miR-200c as compared to ZEB1.
